# Preparation of multigradient hydroxyapatite scaffolds and evaluation of their osteoinduction properties

**DOI:** 10.1093/rb/rbac001

**Published:** 2022-02-01

**Authors:** Hao Huang, Anchun Yang, Jinsheng Li, Tong Sun, Shangke Yu, Xiong Lu, Tailin Guo, Ke Duan, Pengfei Zheng, Jie Weng

**Affiliations:** 1 Key Laboratory of Advanced Technologies of Materials (MOE), School of Materials Science and Engineering, Southwest Jiaotong University, Chengdu 610031, P.R. China; 2 Engineering Laboratory of Orthopedic Implant Device Development and Application Technology, Clinical College of Medicine, Southwest Medical University, Luzhou 646000, P.R. China; 3 Department of Orthopaedic surgery, Children’s Hospital of Nanjing Medical University, Nanjing 210008, P.R. China

**Keywords:** porous hydroxyapatite, scaffold, biocompatibility, osteoinduction, bone tissue engineering

## Abstract

Porous hydroxyapatite (HA) scaffolds are often used as bone repair materials, owing to their good biocompatibility, osteoconductivity and low cost. Vascularization and osteoinductivity of porous HA scaffolds were limited in clinical application, and these disadvantages were need to be improved urgently. We used water-in-oil gelation and pore former methods to prepare HA spheres and a porous cylindrical HA container, respectively. The prepared HA spheres were filled in container to assemble into composite scaffold. By adjusting the solid content of the slurry (solid mixture of chitin sol and HA powder) and the sintering temperature, the porosity and crystallinity of the HA spheres could be significantly improved; and mineralization of the HA spheres significantly improved the biological activity of the composite scaffold. The multigradient (porosity, crystallinity and mineralization) scaffold (HA-700) filled with the mineralized HA spheres exhibited a lower compressive strength; however, *in vivo* results showed that their vascularization ability were higher than those of other groups, and their osteogenic Gini index (Go: an index of bone mass, and inversely proportional to bone mass) showed a continuous decrease with the implantation time. This study provides a new method to improve porous HA scaffolds and meet the demands of bone tissue engineering applications.

## Graphical Abstract

**Figure:**
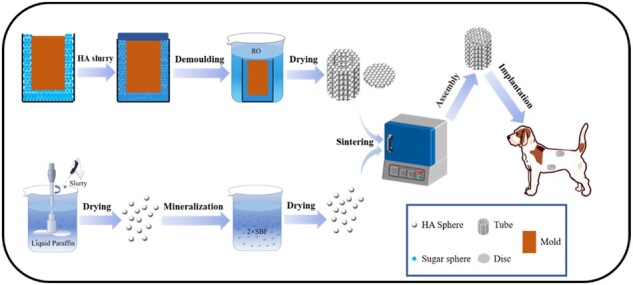

## Introduction

Large bone defects caused by traumas, accidents, congenital deformities, inflammation and tumor resection are difficult to heal spontaneously [1–3]. Autologous bone grafting is the gold standard for the treatment of this condition, but faces problems such as limited graft availability and secondary damage [4]. Other treatment options, such as allografting, are limited by immune rejection [5]. Therefore, synthetic bone substitutes are highly needed. These products should have satisfactory biocompatibility, osteoconductivity, osteoinductivity and mechanical properties matching those of human bone [6]. Hydroxyapatite [HA, Ca_10_(PO_4_)_6_(OH)_2_] is the main inorganic component of human hard tissues, and porous HA scaffolds are widely used in the repair of large bone defects [7, 8]. It is well established that blood vessels play an important role in the repair of bone defects [9, 10]. However, the inner pores of a HA scaffold are located away from the surrounding tissues, which creates a barrier to nutrient transport and blood vessel infiltration into these pores, adversely affecting bone ingrowth into the scaffold [11]. Therefore, improving the angiogenesis and osteogenesis inside a porous HA scaffold is a crucial task to enhance its bone formation ability. Several studies reported that constructing a hollow channel in a porous scaffold improved the oxygen/nutrient perfusion, enhanced the degree of vascularization of the porous scaffolds and promoted bone ingrowth [11, 12]. However, the introduction of such channels also impaired the compressive strength of the scaffold [13–15] as well as cell attachment and proliferation, negatively affecting angiogenesis and osteogenesis. Zhang *et al*. fabricated a porous silicon-based scaffold containing hollow-pipe structures and bioactive ions by the 3D printing technology, and discovered that the hollow-pipe structures and bioactive ions synergistically promoted the internal vascularization of the porous scaffold, ultimately enhancing its bone formation ability [16].

HA prepared in the form of spheres showed excellent performance in bone tissue engineering [17]. Moreover, the introduction of porous structures in HA spheres improved their drug delivery efficiency and osteogenic response [18]. In addition, HA spheres could upregulate the expression of angiogenesis-related genes [17]. Although the osteoconductivity and osteoinductivity of HA are well established, it’s *in vivo* degradation is too slow compared to the rate of osteogenesis; this mismatch hinders the replacement of the scaffold by newly formed bone [19]. The degradation rate of HA is closely related to its crystallinity [20]; in particular, adjusting the crystallinity of HA not only accelerates its degradation, but also improves its mechanical properties [21]. Various strategies have been adopted to improve the osteoinductivity of HA scaffolds. For example, a common method involves loading osteogenesis-related growth factors on the scaffold [22]. However, the use of these factors presents disadvantages such as instability, immunogenicity and high costs [2]. Another effective method to improve the osteoinductivity of a scaffold involves surface modifications, such as coatings [23, 24].

Blood vessels play an important role during bone development and fracture healing, by supplying oxygen, nutrients and cells [10]. Therefore, the enhanced angiogenic ability of porous scaffolds can improve their osteogenic properties [11, 25]. Microporous structures on the scaffold surface can also improve its vascularization ability [26]. For instance, Zhou *et al*. deposited a porous titanium dioxide/calcium-phosphate coating on the surface of a titanium substrate, and showed that the coating enhanced the vascularization and osteogenic properties of the titanium substrate *in vitro* [27]. Therefore, an optimal porous HA scaffold should have satisfactory angiogenesis, osteoinductivity and mechanical properties, as well as a degradation rate matching that of new bone formation. Augmenting a mineralized coating on the surface of the porous scaffolds could enhance the osteogenic effect of the porous scaffold [28]. In order to further improve the angiogenesis and osteogenesis effect of porous scaffolds, especially the angiogenesis and osteogenesis effect inside the porous scaffolds, we introduced the micropores and mineralized coating into porous HA scaffolds in the form of HA spheres. In particular, we prepared porous HA scaffolds by filling the HA spheres in a HA-based container. The latter was fabricated by the pore former method, and the porosity, crystallinity and surface topography of the HA spheres were controlled by varying the processing conditions. The effects of these properties on the vascularization and osteogenesis of the scaffold were evaluated using an intramuscular implantation model. The porosity, crystallinity and coating of the HA spheres and their effect on the vascularization and osteogenesis performance of porous HA scaffolds were systematically investigated.

## Materials and methods

### Preparation of porogen and HA slurry

Canola oil (300 ml, purity level 3) was preheated to 156°C over an oil bath and kept at this temperature for 10 min. Sucrose (70.0 g, purity level 1) was melted at 150°C and rapidly added into the canola oil under stirring (320 rpm). After stirring for 12 min, 600 ml of cold (0°C) canola oil was added to the mixture. Sucrose drops solidified into spheres and were separated with a sieve (mesh size: 1.0–1.2 mm), followed by rinsing repeatedly with *n*-hexane (purity >99%). The obtained spheres were stored in a refrigerator (2°C) before use.

The HA slurry was prepared as follows: anhydrous lithium chloride (LiCl, 5.00 g, AR) was added in 100 ml of *N, N*-dimethylacetamide (DMA, purity ≥ 99.8%) and stirred for 30 min to give a clear solution. Chitin (1.00 g, BR) was dispersed in the solution and magnetically stirred for 12 h to form a yellowish sol. HA powder (HAp: 35.0 g) was added to the sol and stirred for 24 h until a uniform milky white slurry was formed.

### Preparation of HA spheres

#### Sintering

The above slurry was loaded in a syringe and dropwise injected into 400 ml of liquid paraffin (AR) under stirring (300 rpm). After continuous stirring for 2 h, 200 ml of deionized water was slowly added to the mixture and the system was stirred for another 30 min. The liquid paraffin and LiCl were removed by repeatedly rinsing with 60°C deionized water. A silver nitrate assay was conducted until chloride was undetectable in the effluent. The HA spheres were thoroughly dried at 60°C and then sintered at 700 or 1200°C for 2 h (heating rate: 5°C/min). The spheres prepared from 15.0 or 30.0 g of HAp and sintered at 1200°C are labeled S15-1200 and S30-1200, respectively, whereas those obtained from 15.0 g of HAp and sintered at 700°C are denoted as S15-700.

#### Surface coating by immersion in double-concentrated simulated body fluid

Some HA spheres were surface-modified by immersion in double-concentrated simulated body fluid (2×SBF), i.e. a solution containing double concentrations of primary inorganic ions in human serum (Table 1).

**Table 1.** Order of addition, amount, purity and formula weight of reagents used for preparing 500 ml of 2×SBF

Order	Reagent	Amount	Purity (%)	Formula weight
1	NaCl	8.0035g	99.5	58.4430
2	NaHCO_3_	0.355 g	99.5	84.0068
3	KCl	0.225 g	99.5	74.5515
4	K_2_HPO_4_•3H_2_O	0.231g	99.0	228.2220
5	MgCl_2_•3H_2_O	0.311g	98.0	203.3034
6	1mol/L HCl	39 ml	–	–
7	CaCl_2_	0.292g	95.0	110.9848
8	Na_2_SO_4_	0.072g	99.0	142.0428
9	Tris	6.118g	99.0	121.1356
10	1mol/L HCl	0-5ml	–	–

The solution was prepared by following the procedure of Girija *et al*. [29], with the pH adjusted to 7.3. One gram of S15-700 spheres and 8 ml of 2×SBF were added into a 10-ml Eppendorf tube. The tube was maintained in a 37°C water bath for 7 days, refreshing the 2×SBF solution every 12 h. After 7 days, the spheres were collected and denoted as S15-M.

### Preparation of container

The HA container (HAc) was composed of HA tubes and discs, which were prepared by a porogen-based method, as described in the following. The sucrose spheres were added into a lab-made mold, gently shaken and pressed lightly with a piston. The mold was maintained in an oven (70°C) for 8 min to form local connections between spheres. The slurry described in section ‘Preparation of porogen and HA slurry’ was added into the mold and pushed to infiltrate the interstices between the spheres. Anhydrous ethanol (AR) was added to the mold to induce the gelation of DMA. Then, the content of the mold was carefully immersed in deionized water and allowed to rest for 6 h, during which the sucrose spheres (i.e. porogen) were dissolved. The resulting sample was rinsed repeatedly with water, trimmed to the desired shape (when necessary), dried (60°C, 24 h) and then sintered at 1200°C for 2 h.

### Assembly of composite scaffolds

**Figure 1.**
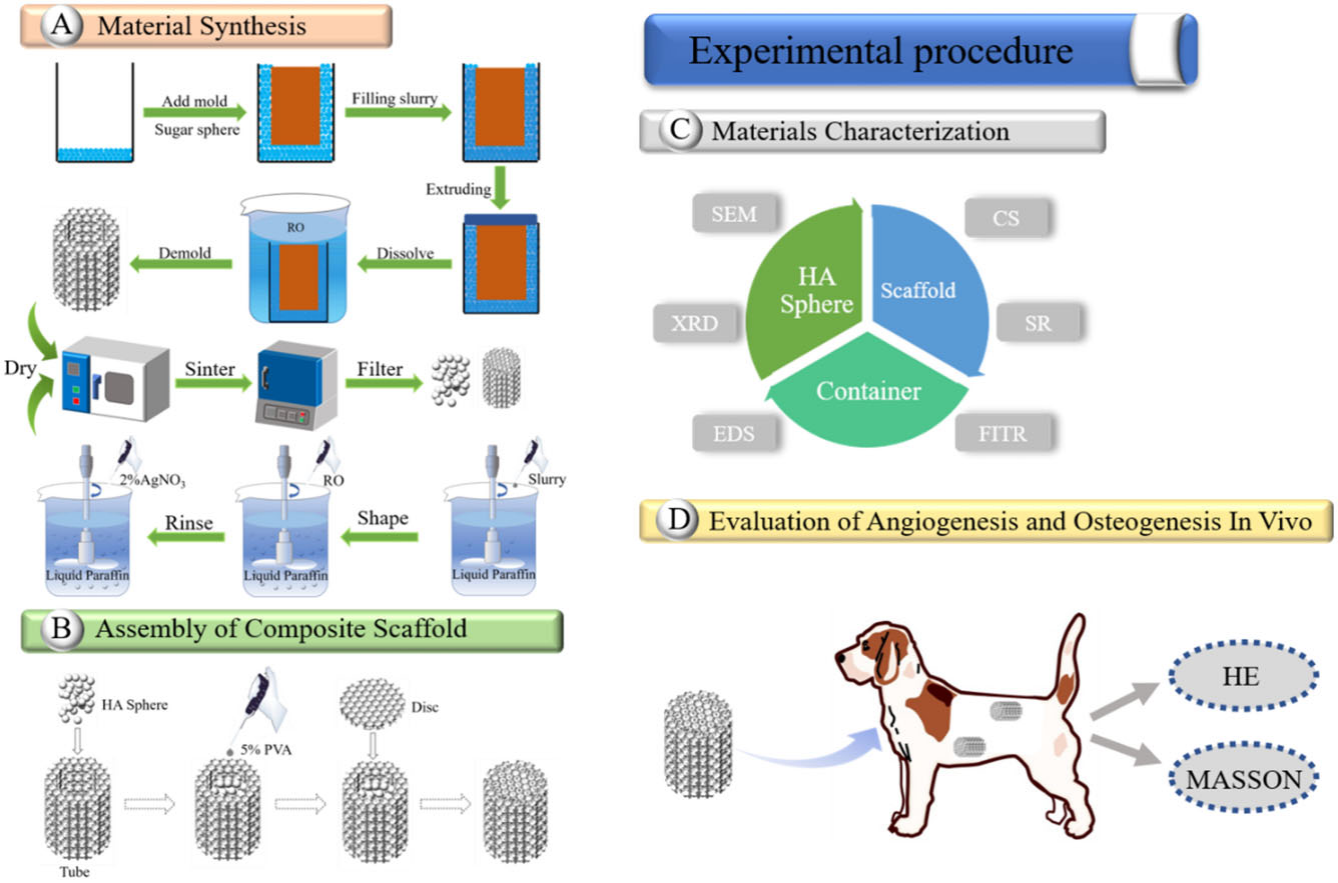
Flow diagrams of experimental procedure

A HA disc was bonded to one end of a tube using 5% wt. polylactic acid (purity >99%) as the adhesive. Then, the HA spheres were filled into the tube and gently vibrated. Finally, the other end of the tube was capped with another HA disc, forming the assembled scaffold (Fig. 1). The scaffolds were labeled as HA30, HA15, HA-700 and HA-M according to the containers filled with S30-1200, S15-1200, S15-700 and S15-M, respectively.

### Physicochemical characterizations

The surface morphology and phase composition were characterized by scanning electron microscopy (SEM; QUANTA200, FEI, USA) and X-ray diffraction (XRD; PANalytical X'Pert, Netherlands; Cu K_α_, 35 mA, 45 kV), respectively. Chemical structures were analyzed by Fourier transform infrared spectroscopy (FTIR; Thermo Electron Nicolet 5700, USA; 400–4000 cm^−1^). Dimensional changes during processing (i.e. drying, sintering) were measured with a caliper. The compressive strengths of HA spheres and composite scaffolds were measured on a universal testing machine (Instron 5567, USA; 0.5 mm/min), the HA spheres and the composite scaffolds were placed in an oven (110°C) to dry for 2 h and then cooled at room temperature for 30 min before testing (*n* = 6). The porosities of the HA scaffolds were determined by Archimedes’ principle. Briefly, three scaffolds were placed in a 10-ml graduated cylinder containing 5.00 ml of water. The cylinder was transferred in a vacuum oven, which was evacuated (< 100 Pa) for 2 h and then released to push water into the accessible pores. Then, the porosity was calculated as:

(1) $$V=\sum_{x=1}^{3}\left(\frac{\pi D_{x}^{2}}{4}\times H_{x}\right);P=\left[\frac{V-V_{1}+5}{V}\right]\times 100\%,$$

where *D_x_* and *H_x_* are the diameter and height of the HA scaffolds, respectively, *x* represents the *x*-th scaffold sample, *V* is the total volume of the three scaffolds and *V*_1_ is the reading of the vacuum oven [30].

### *In vivo* model

Six 10-month-old male beagle dogs (weight: 11–13 kg; purchased from Sichuan Provincial People’s Hospital) were randomized into three groups according to the period of study planned: 4, 12 and 24 weeks. Two sites were used for implantation in each animal: the abdominal cavity and dorsal muscles. The HA30 and HA-M scaffolds were implanted in the former group, while the HA15, HA30, HA-700 and HA-M scaffolds were implanted in the latter. This study and all protocols were approved by the Research Ethics Committees of both Southwest Jiaotong University and Sichuan Provincial People’s Hospital.

The animals were anesthetized by intravenous injection of sodium pentobarbital (3 mg/kg). Eight incisions (length: 3 cm) were made bilaterally 3 cm from the dorsal midline, and eight muscle pockets were formed. After placing the scaffolds in the pockets, the wound was sutured layer by layer. Subsequently, four incisions were made bilaterally in the abdominal region, at 3 cm lateral to the groin. The skin, underlying muscle and peritoneum were separated, and the scaffolds were sutured to the peritoneal wall with non-degradable threads (2-0; Yangzhou Huanyu). Finally, the wound was closed.

At three time points (4, 8 and 12 weeks after operation; *n *=* *2), animals were killed by overdose intravenous injection of pentobarbital sodium. The scaffolds were harvested and fixed in 4% paraformaldehyde for 7 days, then rinsed with deionized water for 24 h. After that, the samples were decalcified by 10% EDTA solution for 4 weeks, rinsed with deionized water and dehydrated with 70%, 80%, 90%, 95% and 100% gradient ethanol. Finally, the samples were embedded in paraffin and cut into sections longitudinal to the central part (thickness: 5 µm). The sections were stained alternately with hematoxylin–eosin (HE) and Masson’s trichrome reagents on successive sections. The microstructure and morphology of the sections were observed using an inverted optical microscope; the area percentage of new bone and the number of blood vessels in the samples were analyzed using the ImageJ software (National Institutes of Health, America).

### Statistical analysis

Statistical analyses were carried out using SPSS v16 software (IBM Corp., Armonk, NY, USA), and all data were shown as means ± SD. For the comparison among three or more groups, Kruskal–Wallis H test was used; if significant difference was detected, pairwise post-hoc comparison using Mann–Whitney tests with Bonferroni correction was followed and two-way t test was used in experiments with two groups at each time point. For all tests, *P* < 0.05 was considered statistically significant.

## Results and discussion

### Morphology of HA spheres

**Figure 2.**
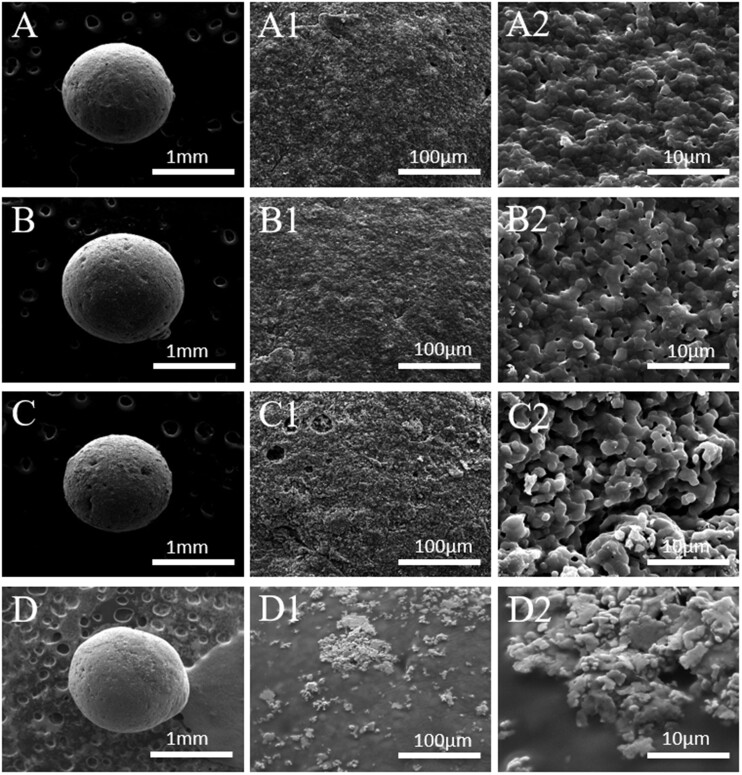
SEM images of HA spheres: S30-1200 (**A**, **A1**, **A2**), S15-1200 (**B**, **B1**, **B2**), S15-700 (**C**, **C1**, **C2**) and S15-M (**D**, **D1**, **D2**)

**Figure 3.**
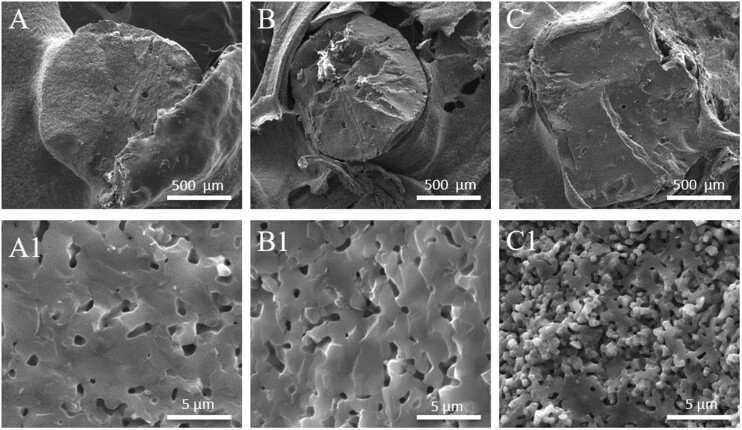
Cross-section SEM images of HA spheres: (**A**, **A1**) S30-1200, (**B**, **B1**) S15-1200 and (**C**, **C1**) S15-700

As shown in Fig. 2A–D, all HA spheres had good sphericity and small pores were observed on their surface; S30-1200 showed fewer pores than S15-1200, owing to its higher HA content. Moreover, the pores of S15-700 were larger than those of S15-1200, but their pore numbers were not significantly different; this may be related to the higher sintering temperature of S15-1200, which led to a more complete grain development and to fewer grain boundary defects [31]. S15-700 showed large and unevenly distributed pores, as shown in Fig. 2C–C2; in addition, the surface roughness of S15-700 was higher than that of S30-1200 and S15-1200; Fig. 2A2–C2 shows that the grain sizes of S30-1200, S15-1200 and S15-700 were similar, but their pore densities were significantly different, and followed the order S30-1200 < S15-1200 < S15-700. Figure 3 shows cross-section SEM images of S30-1200, S15-1200 and S15-700. Figure 3A–C reveals that micropores of different sizes were distributed inside S30-1200, S15-1200 and S15-700, while S15-700 displayed the largest number of pores. S15-700 exhibited the smallest internal grain size (Fig. 3A1–C1), while the grain sizes of S30-1200 and S15-1200 were similar; the internal porosity of S15-1200 was slightly greater than that of S30-1200, while S15-700 had significantly higher porosity than S30-1200 and S15-1200. It is worth noting that the internal grain size of S15-700 was smaller than the external one, while internal the grain sizes of S30-1200 and S15-1200 did not change much. In summary, the porosity, grain size and surface roughness of the HA spheres were controlled by the initial content of HA slurry and the sintering temperature.

### *In vitro* mineralization of HA spheres

**Figure 4.**
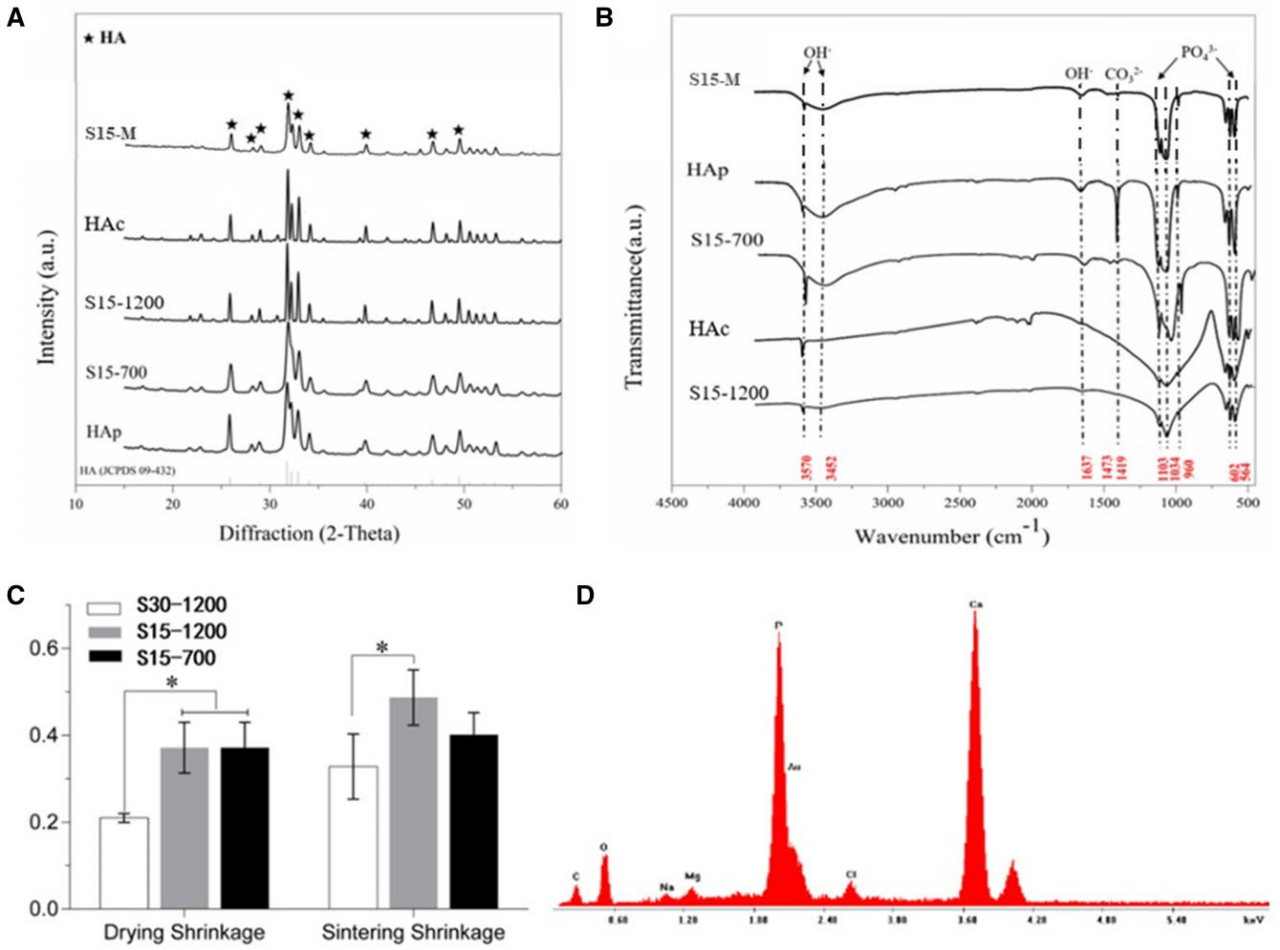
(**A**) XRD patterns and (**B**) FTIR spectra of HAp, HA spheres and HA container; (**C**) drying and sintering shrinkages of HA spheres; (**D**) surface elemental analysis of S15-M. *P <* *0.05 indicates statistically significant differences

Figure 2D shows that the distribution of pores on the surface of S15-M was less pores than that of other groups, and its pore size was smaller; this was due to the mineralized coating covering the pores of S15-M. Figure 2D1 and D2 reveals that the mineralized coating distributes on the surface of S15-M evenly, whereas the coating did not completely cover the surface of S15-M. The energy-dispersive spectroscopy analysis of the mineralized coating is shown in Fig. 4D; calcium and phosphorus were the main elements contained in the coating, along with small amounts of magnesium and sodium. Previous related studies have shown that the mineralization of the biomaterial surface could increase the rate of bone formation [32, 33].

### Phase and chemical structure analysis of composite scaffolds

The XRD patterns of HAp, S15-700, S15-1200 and HAc are shown in Fig. 4A. The characteristic peaks of HA are marked with a black star symbol; all peaks corresponded to the standard JCPDS card of HA (09-432), indicating that all samples consisted of HA phases without impurities [34]. As shown in Fig. 4A, the crystallinity of S15-1200 and HAc was significantly higher than that of HAp and S15-700; however, the full width at half-maximum of S15-700 was similar to that of HA, and its crystallinity was lower.

The XRD results indicate that the molding procedure and three-dimensional structure (spherical of HA spheres and closed barrel shape of container) had little effect on the degree of crystallinity of the samples, which was mainly determined by the sintering temperature. The FTIR spectra of HAp, S15-700, S15-1200 and HAc are shown in Fig. 4B. All samples showed the characteristic peaks of HA, i.e. the bending peaks of P-O in ${\text{PO}}_{4}^{3-}$ groups at 564 and 602 cm^−1^ and the tensile vibration peaks at 958, 1034 and 1108 cm^−1^, with no impurity peaks [35]. ${\text{CO}}_{3}^{2}$^–^ groups appeared at 1419 cm^−1^ could be attributed to HAp was easy to form Type-B carbonated HA when exposed to the air, but with high temperature sintering, the carbonate will be gradually removed [36]. After the same treatment of all samples, peaks corresponding to H-O tensile and bending vibrations appeared at 3570 cm^−1^ (belonging to the 3700–2500 cm^−1^ band) and 1639 cm^−1^ were belonged to the characteristic peaks of HA. The H-O characteristic peaks of S15-700, S15-M and HAp at 1639 cm^−1^ were more evident, which may be the result of sintering at high temperature has a tendency to eliminate the functional group OH in the HA matrix (dehydration) [37]. The characteristic peaks of S15-700 and S15-M were consistent with those of the HAp, indicating that chitin was completely removed and main component of the mineralized phase was HAp after sintering and mineralization, respectively. Furthermore, the stretching vibration of C-H are around 2900 cm^−1^ and 1380 cm^−1^, and deformation vibration of C-H is around 1430 cm^−1^, respectively, which were disappeared in characteristic peaks of S15-700. These data demonstrated that DMA has all disappeared and the sintering has been completed.

### Analysis of composite scaffold morphology and container porosity

**Figure 5.**
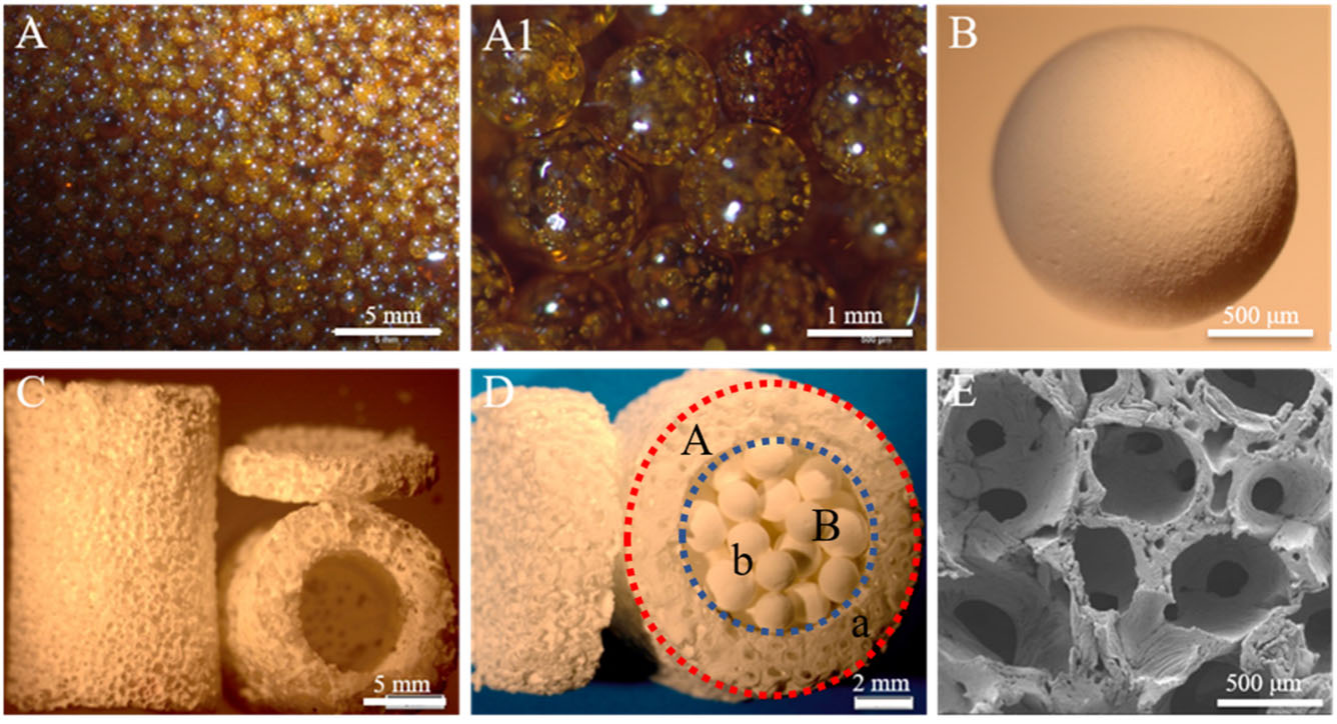
Optical and SEM images of (**A**, **A1**) sucrose spheres, (**B**) HA spheres, (**C**, **E**) HA container and (**D**) composite scaffold

Figure 5A shows that the size of the sugar spheres was ∼ 1.2 mm. After being kept at 70°C for 8 min, bridging necks were formed between these spheres (Fig. 5A1); the HA spheres obtained after sintering are shown in Fig. 5B. Figure 5C and E shows that the sizes of the macropores and interconnecting pores were 1 mm and 150–300 µm, respectively, indicating a good penetration of the spheres in the container. The highly interconnected porous structure facilitates nutrient transport inside the scaffolds and host fiber vascularization [38]. Figure 5D shows that the composite HA scaffold had an outer diameter of 10 mm, a height of 15 mm, an inner diameter of 6 mm and a porosity of ∼ 80% (Table 2). A previous study reported that a scaffold pore size and porosity reaching 120 µm and 80%, respectively, were beneficial for the differentiation of osteoblasts [39]. To sum up, the structure of the prepared HA scaffolds facilitated angiogenesis and osteoblast differentiation.

**Table 2.** Shrinkage rate of container and the porosity of composite scaffold

Original size (mm)		Sintered size (mm)		Shrinkage (%)		Porosity (%)
H	D	H	D	H	D	–
14.0	19.0	7.11 ± 0.22	10.24 ± 0.13	50.71 ± 1.57	53.72 ± 0.69	79.2 ± 1.9

### Shrinkage rate and mechanical properties of HA spheres and composite scaffolds

Figure 4C shows that the drying shrinkage rates (SR) of S30-1200, S15-1200 and S15-700 were inversely proportional to the HA content, namely, S30-1200 < S15-1200; the drying shrinkage of S30-1200 was 20.96%, while those of S15-700 and S15-1200 were both 37.14%. The sintering shrinkage rate was proportional to the sintering temperature, namely, S15-700 < S15-1200; in particular, the sintering shrinkage rate of S15-700 and S15-1200 were 40.10% and 48.69%, respectively; moreover, the rates showed a trend inversely proportional to the HA content, namely, S30-1200 < S15-1200, and the rate of S30-1200 was the lowest (32.77%). Therefore, the sizes of macropores and interconnecting pores of the container could be regulated by varying the size of the sugar spheres and the time/temperature conditions of their heat treatment, respectively. Table 2 shows that the transverse and longitudinal SR of the container were similar, demonstrates that the overall architecture of the container has not collapsed and the porosity of composite HA scaffold is close to 80%, which can promote scaffold–tissues/cells interactions [40].

The compressive strengths of the HA spheres and the two groups of composite scaffolds are shown in Table 3. The compressive strength of the HA spheres increased in the order S15-700 < S15-M < S15-1200 < S30-1200. After mineralization and coating, the compressive strength of S15-700 increased from 0.46 to 0.61 MPa, whereas that of S15-1200 increased by four times (2.34 MPa) after sintering at 1200°C, and that of the S30-1200 was 3.51 MPa, which was 50% higher than that of S15-1200. These data indicate that increasing the sintering temperature and HA content as well as adding the mineralized coating increased the compressive strength of the HA spheres. The compressive strengths of HA30 and HA-M were 1.67 and 1.12 MPa, respectively, and the 50% increase indicates that S30-1200 inside the scaffold bore part of the pressure; the compressive strength of HA-M was higher than that of S15-M, indicating that the scaffold was the main load-bearing component of HA-M; finally, the compressive strength of HA30 was lower than that of S30-1200, implying that the compressive strength of S30-1200 was higher than that of the scaffold.

**Table 3.** Compressive strength of composite scaffolds and HA spheres

Sample	HA30	HA-M	S30-1200	S15-1200	S17-700	S15-M
Compressive strength/Mpa	1.67 ± 0.23	1.12 ± 0.19	3.51 ± 0.15	2.34 ± 0.27	0.46 ± 0.09	0.61 ± 0.13

### *In vivo* structural changes of composite scaffolds

The ectopic implantation model was employed in this study, as heterotopic models can provide a better description of the osteoinduction ability of the porous HA scaffold [41]. Relevant studies have shown that the osteogenesis effect of heterotopic implantation in dogs was more significant, and osteogenesis effect of the dorsal muscles was better than abdominal cavity [42, 43]. Moreover, the stress load of the dorsal muscles is higher than abdominal cavity, which facilitates comparison of the effects of different stress loads on the structure of the implant material [42]. Therefore, dorsal muscles and abdominal cavity were selected as the research model.

**Figure 6.**
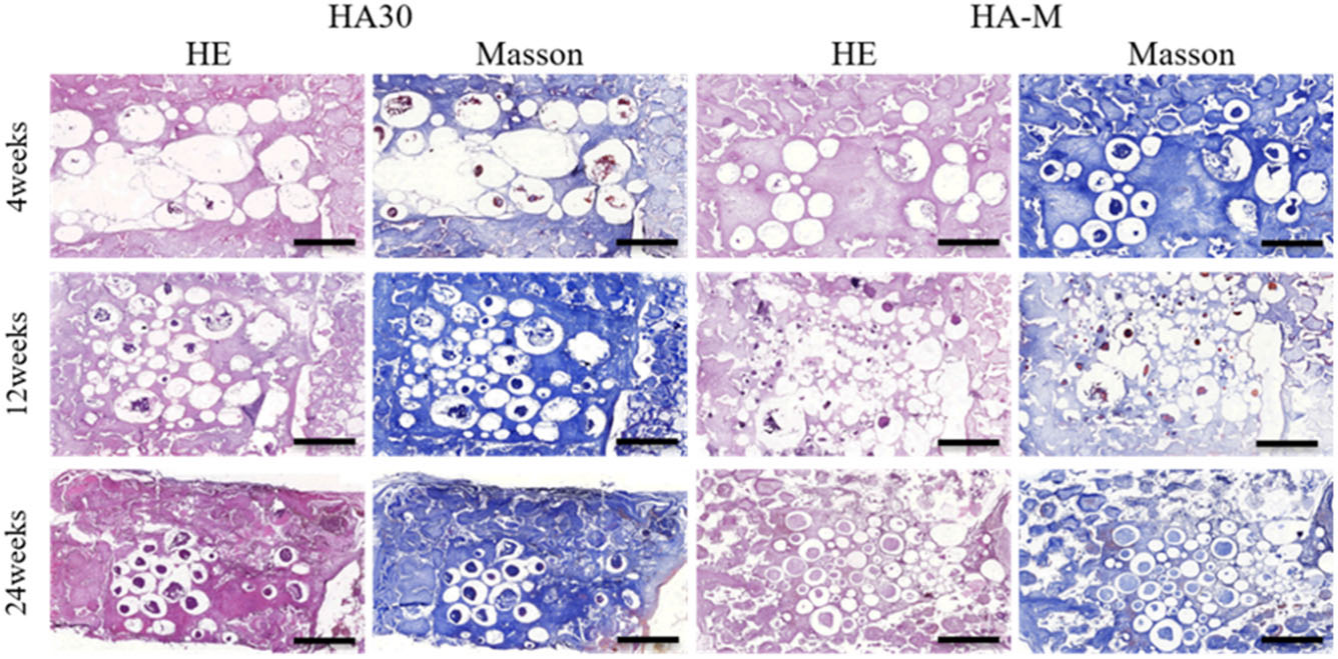
Masson’s trichrome and HE staining images of implant histological sections in abdominal cavity, showing scaffold changes and new bone formation at 4, 12 and 24 weeks post-operation; scale bars = 2 mm

**Figure 7.**
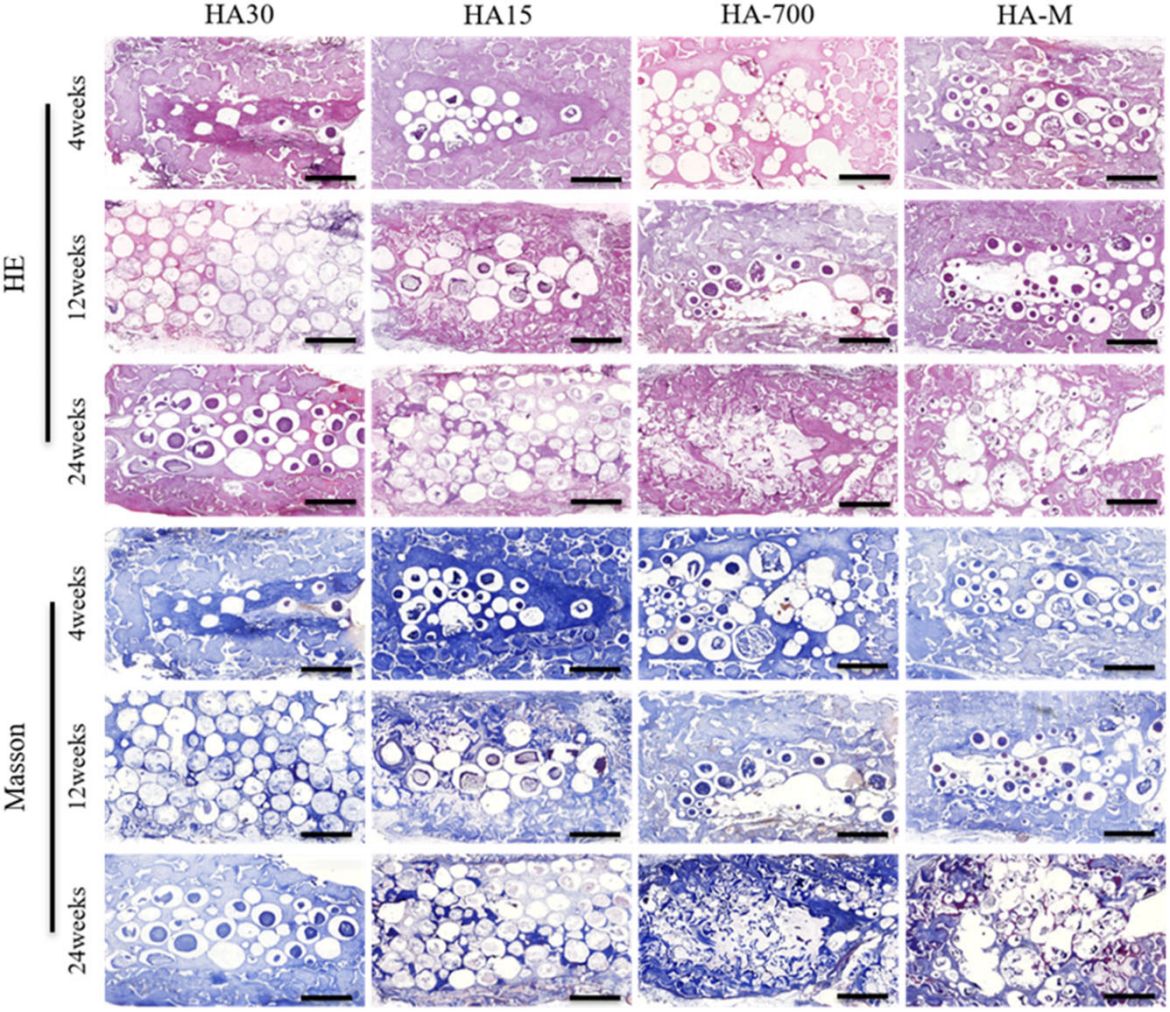
Masson’s trichrome and HE staining images of implant histological sections in dorsal muscle, showing scaffold changes and new bone formation at 4, 12 and 24 weeks post-operation; scale bars = 2 mm

The structural changes of the HA composite scaffolds were evaluated by the degree of deformation of the HAc and the degradation of the HA spheres. Figure 6 shows that the two groups of scaffolds did not show large-scale deformations, because the scaffolds were not compressed in the abdominal cavity. Growth of fibrous tissue, compression of new bone tissue and body fluid corrosion led to the fragmentation of S15-M inside HA-M. The compression resistance and low crystallinity of S15-M are two additional factors that can cause its fragmentation in the body; however, the S30 spheres inside HA30 did not break even after 24 weeks. As shown in Fig. 7, all implanted scaffolds exhibited deformations at 24 weeks, with HA-700 and HA-M showing more obvious changes compared to the others. Moreover, the HA spheres inside the HA-700 and HA-M scaffolds collapsed at 12 weeks, and degradation occurred at 24 weeks, due to muscle squeezing; however, the HA spheres inside the HA15 and HA30 scaffolds did not break after 24 weeks. This suggests that the ceramic scaffold was deformed rather than broken in this case, indicating that the growth of soft tissue changed the deformation mode of the ceramic scaffold and improved its mechanical properties [44].

### *In vivo* angiogenesis ability of the composite scaffolds

#### Evaluation of ectopic abdominal cavity vascularization

**Figure 8.**
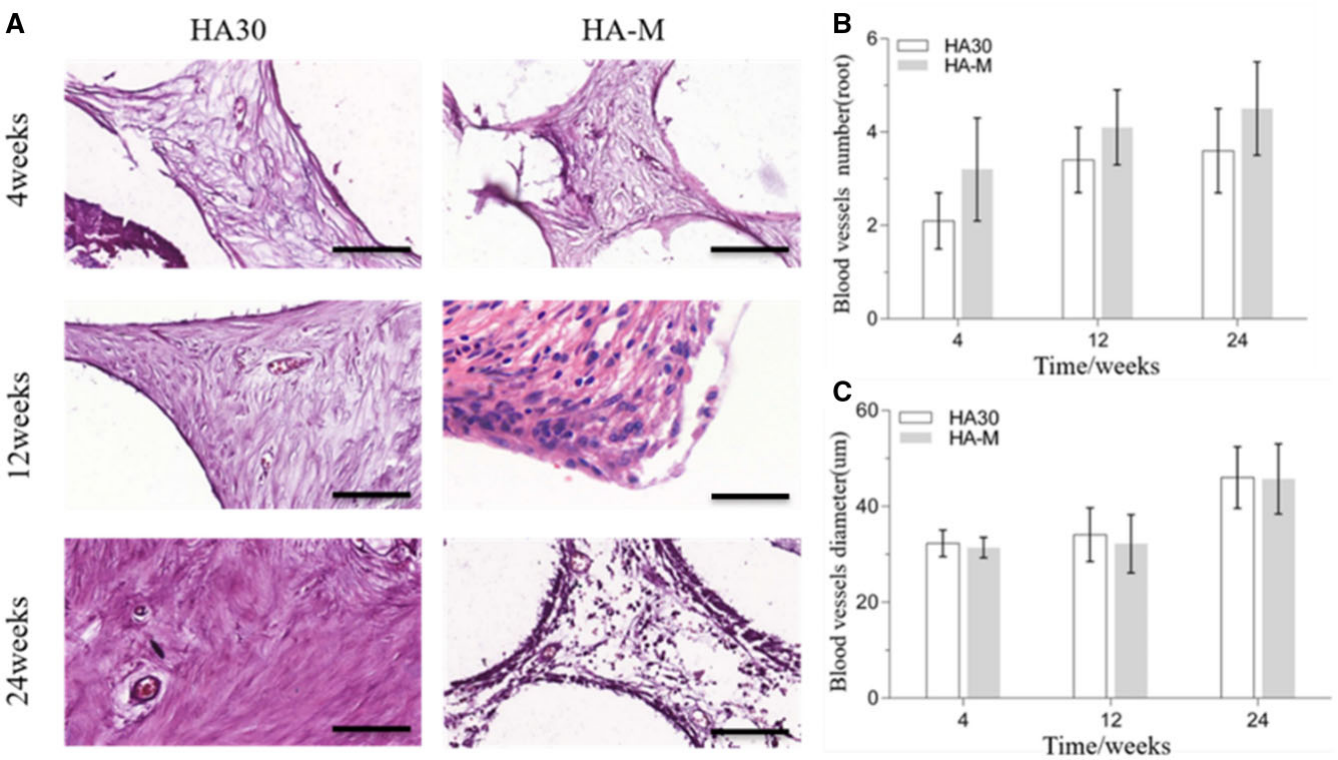
(**A**) HE staining images of blood vessels in scaffolds implanted in abdominal cavity at 4, 12 and 24 weeks (scale bars = 100 μm); (**B**) number of blood vessels; (**C**) diameter of blood vessels

HE staining images as well as the number and diameter of blood vessels were used to evaluate the vascularization ability of the implanted scaffolds *in vivo*. Figure 8A shows HE staining section images of the blood vessels inside the scaffold. The blood vessel area inside HA30 and HA-M increased with the implantation time. From the 4th to the 12th week, the number of blood vessels per unit area (mm^2^) inside the two scaffolds increased significantly: from 2.1 to 3.4 for HA30, and from 3.2 to 4.1 for HA-M, with corresponding increase ratios of 61.9% and 28.1%, respectively, as shown in Fig. 8B. Between the 12th and the 24th week, although the number of blood vessels per unit area of the two groups of scaffolds increased by 0.2 and 0.4, respectively, the diameter of the blood vessels also increased significantly; however, there was no significant difference in blood vessel number between the two groups at three time points. In particular, the diameter of the blood vessels inside HA30 and HA-M increased from 34.1 to 46.0 µm and from 32.2 to 45.7 µm, respectively, with increase ratios of 34.9% and 41.9%, respectively, as shown in Fig. 8C. During the same interval, the number of blood vessels in HA-M was much higher than that in HA-30, but there was no significant difference in blood vessel diameter between the two groups, showing that the internal and external structure of the multifactor gradient scaffold was beneficial for the growth of blood vessels.

#### Evaluation of ectopic back muscle vascularization

**Figure 9.**
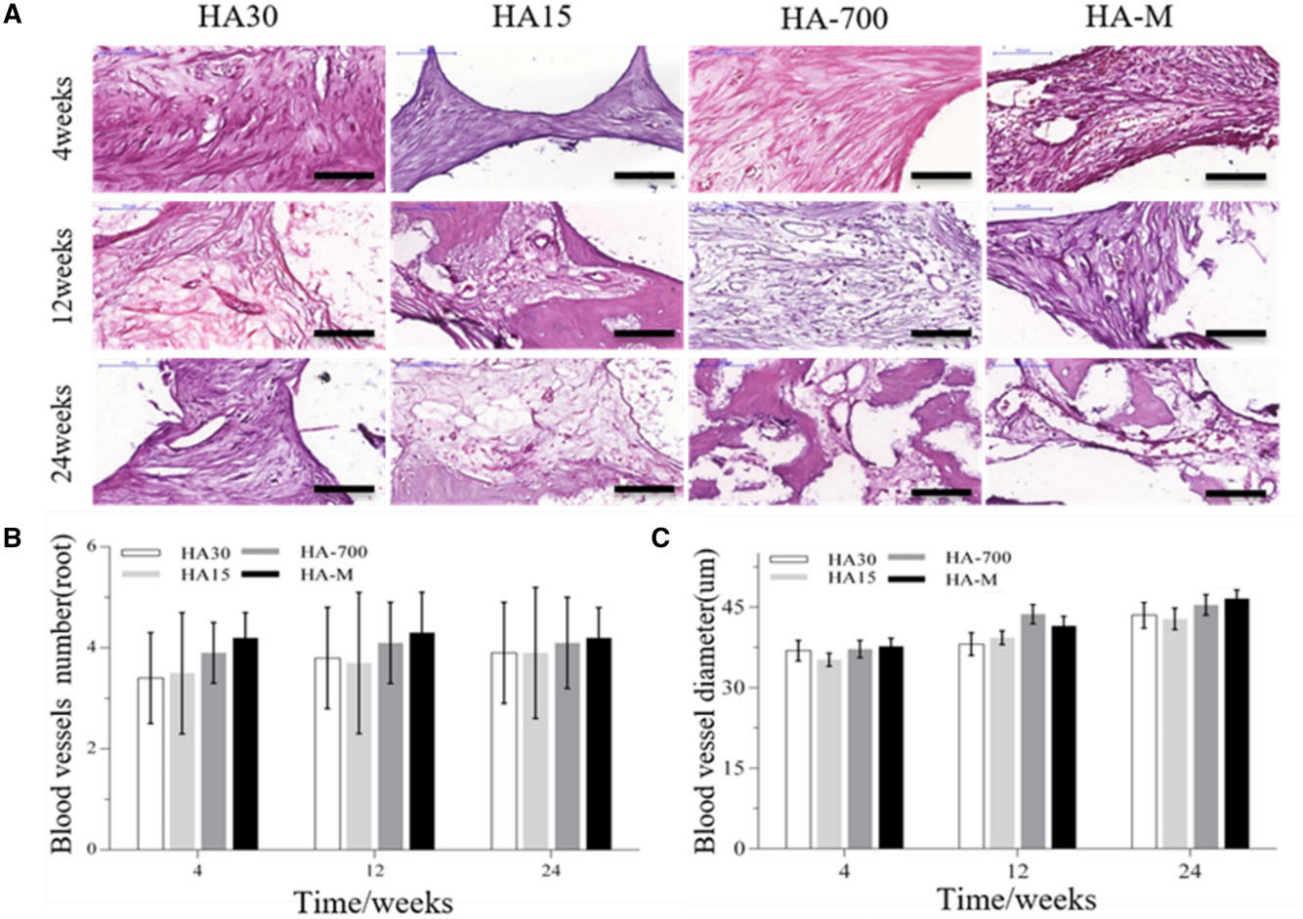
(**A**) HE staining images of blood vessels in scaffolds implanted in back muscles at 4, 12 and 24 weeks (scale bars = 100 μm); (**B**) number of blood vessels; (**C**) diameter of blood vessels. *P <* *0.05 indicates statistically significant differences

HE staining section images of the blood vessels inside the scaffolds are shown in Fig. 9A, while the numbers and diameters of blood vessels are plotted in Fig. 9B and C, respectively. Figure 9B shows that the amount of blood vessels in HA-M did not change with the implantation time, whereas this parameter did increase for the other groups. However, at each observation time, the amount of blood vessels was higher in HA-M than in other groups, whereas HA30 and HA15 showed the lowest values, with no significant difference between them. As shown in Fig. 9C, the diameter of blood vessels inside all implanted scaffolds increased with the implantation time; the angiogenesis ability of HA-M was best; however, there was no significant difference between HA-M and HA-700, but their values were higher than those of HA30 and HA15, which were consistent with the conclusion that HA spheres with low crystallinity and mineralized coatings facilitated the development and ingrowth of blood vessels [45].

Figures 8A and 9A confirm that the vascular wall of HA30 and HA-M was significantly thicker at 24 than at 4 and 12 weeks, indicating that the vascular maturity gradually increased with the implantation time. The results of Figs 8 and 9 show that in the early stage of vascular repair (namely, at 4 weeks), HA-M was much more effective than HA30 in promoting vascular growth. These results show that HA-M had the highest angiogenesis activity, and the multifactor gradient structure enabled the rapid growth and maturation of blood vessels. The low crystallinity, small crystal size, poor mechanical properties and high degradability of the sintered HA spheres markedly facilitated the growth of blood vessels in the scaffold. Although the mineralized coating and reduced HA content could accelerate angiogenesis in the scaffold, their effect was not as prominent as that of the sintering temperature.

### *In vivo* osteogenic ability of composite scaffolds

#### Evaluation of ectopic abdominal osteogenesis

**Figure 10.**
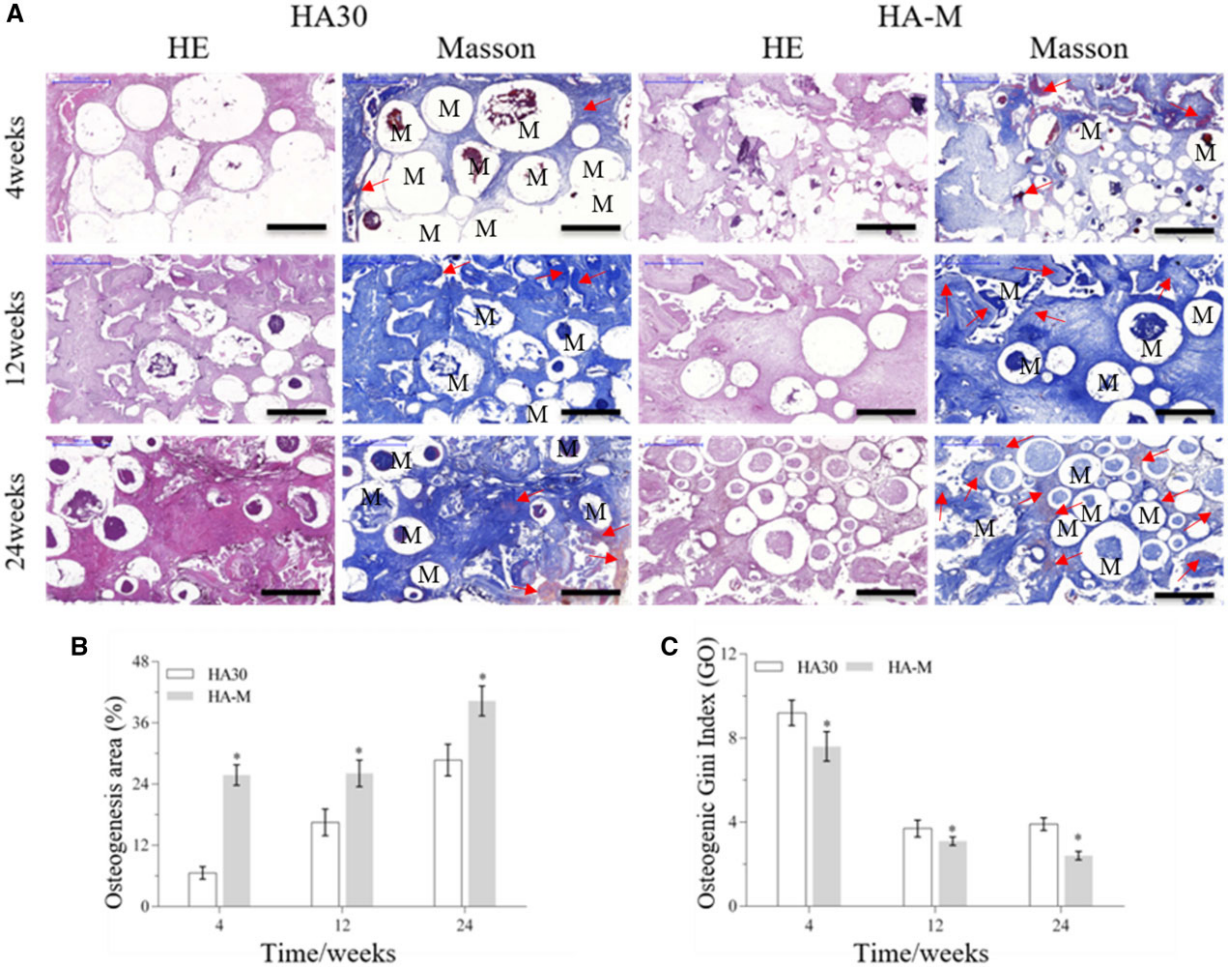
Bone regeneration activity of scaffolds at 4, 12 and 24 weeks after implantation in abdominal cavity. (**A**) HE staining and Masson’s trichrome images (scale bars = 1 mm), red arrow: new bone, M: materials; (**B**) osteogenesis area; (**C**) osteogenic Gini index. *P <* *0.05 indicates statistically significant differences

The *in vivo* osteogenic ability of the implanted scaffolds was evaluated by HE and Masson’s trichrome staining, as well as osteogenic area percentage [osteogenesis area (%) = (T – C – M)/(T); T: whole MASSON staining image; C: collagen area(blue); M: material area(white)] and Gini index [Go= (a × B)/(A × b); A: cross-sectional area of the container; a: osteogenic area of the cross-sectional area of the container; B: internal cross-sectional area of the scaffold, b: osteogenic area of the internal cross-sectional area of the scaffold; which are indicated in Fig. 5D] measurements. Staining images of tissue sections of the internal and external regions of the scaffold are shown in Fig. 10A. Figure 10B and C display the osteogenic area and Go values, respectively. As shown in Fig. 10A and B, the maturity and osteogenesis area of new bone formed outside and inside the scaffold increased with the implantation time. Figure 10C shows that the Go values inside and outside the scaffold dropped significantly from the 4th to the 12th week; the Go values of HA30 and HA-M decreased from 9.2 to 3.7 and from 7.6 to 3.1, with decrease ratios of 59.8% and 59.2%, respectively. However, from the 12th to the 24th week, the Go values of HA30 increased by 0.2 and HA-M decreased by 0.7, with a decrease ratio of 22.6%. In the same period, a significantly higher proportion of bone area was observed in HA-M than HA30, and the Go value of HA-M was lower than that of HA30. Furthermore, the Go value of HA30 showed no downward trend from the 12th to the 24th week, while HA-M continued to decline. These results suggest that HA-M had superior ectopic abdominal osteogenesis ability to HA30.

#### Evaluation of ectopic back muscle osteogenesis

**Figure 11.**
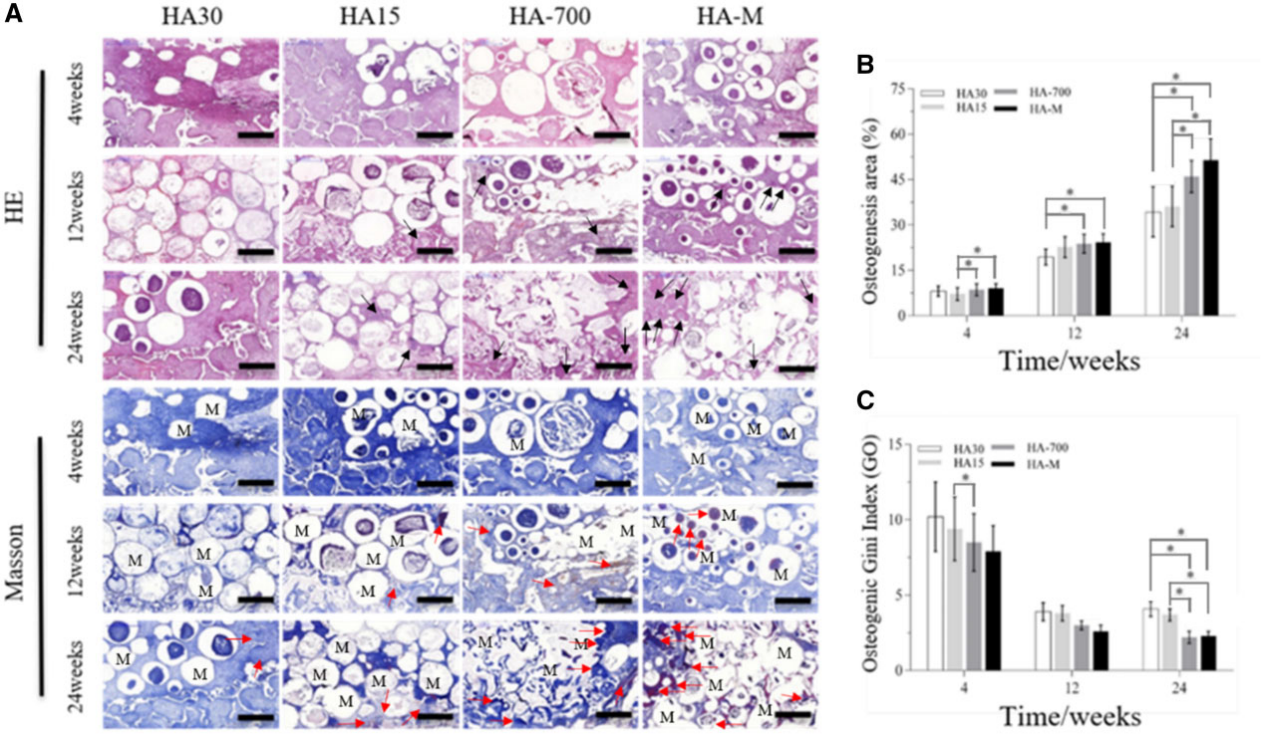
Bone regeneration activity of scaffolds at 4, 12 and 24 weeks after implantation in dorsal muscles. (**A**) HE staining and Masson’s trichrome images (scale bars = 1 mm), black arrow: Havers systems, red arrow: new bone, M: materials; (**B**) osteogenesis area; (**C**) osteogenic Gini index. *P <* *0.05 indicates statistically significant differences

Staining images of internal and external tissue sections of implanted scaffolds are shown in Fig. 11A, while Fig. 11B and C show the osteogenesis area and Go values, respectively. Figure 11A and B reveals that the maturity and osteogenesis area of new bone formed in the implanted scaffolds increased with the implantation time; the osteogenic effects were more marked for HA-700 and HA-M, with a large area corresponding to lamellar bone and Havers’ systems. At the 12th and 24th week, there was significant differences between HA-M and HA30, and HA-700 and HA30 also showed significant differences. Figure 11C shows that the Go values of all implanted scaffolds decreased significantly from the 4th to the 12th week. In particular, the Go values decreased from 10.2 to 3.9 (HA30), from 9.4 to 3.8 (HA15), from 8.0 to 3.0 (HA-700) and from 7.9 to 2.6 (HA-M), with corresponding decrease ratios of 61.8%, 59.6%, 64.7% and 67.1%, respectively. However, from the 12th to the 24th week, the Go value of HA30 increased by 0.2, while those of the other groups decreased; in particular, the Go value of HA-700 showed the largest drop (0.8), with a decrease ratio of 26.7%. At the 4th and 12th week, the Go value of HA-M was the lowest, whereas HA-700 had the lowest Go value at the 24th week, and no significant difference was observed between the Go values of HA-M and HA-700; however, there were significant differences between HA-M and HA30, as well HA15. The Go values followed the order HA30 > HA15 > HA-700 > HA-M. From the 12th to the 24th week, the Go value of HA30 showed no downward trend, in contrast with the other groups. At each time point, HA-M showed the largest new bone area, whose values decreased in the order HA-M > HA-700 > HA15 > HA30.

The new bone area data show that the osteopromotive effects of HA-M were significantly higher than those of HA30, which was consistent with the blood vessel growth data. Especially in the early stages of osteogenesis, HA-M could facilitate rapid formation of new bone, confirming that the multifactor gradient structure resulted in a faster generation of new bone. Figure 11B confirms that the sintering and *in vitro* coating processes were beneficial for the regeneration of bone tissue; the bone-like apatite layer resulted in an increased rate of bone formation, while factors such as low crystallinity and high porosity increased the amount of formed bone. Between the 12th and 24th week, the Go value of HA30 in the abdominal cavity and back muscles did not continue to decrease and reached a value of 4, indicating that the osteogenesis areas inside and outside the scaffold were unbalanced; however, the Go values of HA-700 and HA-M continued to decrease, indicating an overall balanced bone formation across the scaffold. The Go value of HA-M decreased rapidly at the 4th week, consistent with the blood vessel growth data. These results show that HA-M possessed not only excellent vascularization ability, but also good heterotopic osteogenic activity.

## Conclusion

We prepared several groups of HA spheres and a porous HAc by regulating the HA slurry content and the sintering temperature, as well as adding a mineralized coating. The assembled HA spheres and porous HAc were combined into a multigradient scaffold. Compared with the HA30, HA15 and HA-700 scaffolds, the results of heterotopic *in vivo* experiments demonstrated that HA-M could accelerate the growth of blood vessels and bone formation, rapidly reducing the osteogenic Gini index in the intermediate and final stages of bone remolding. This accelerated the maturation of blood vessels, enhanced the supply of nutrients and oxygen, and improved the osteogenesis, continuing to reduce the osteogenic Gini index. In summary, the multigradient scaffold proposed in this study possessed excellent ectopic vascularization and osteogenesis capabilities, which provides a new solution to the problem of poor internal osteogenesis in large bone defects.

## Author contributions

H.H.: writing—original draft, conceptualization, methodology. A.Y.: methodology, investigation. J.L.: methodology, investigation. T.S.: formal analysis. S.Y.: formal analysis. X.L.: formal analysis. T.G.: formal analysis, visualization. K.D.: formal analysis. P.Z.: supervision, funding acquisition. J.W.: conceptualization, supervision, funding acquisition.

## Funding

This research was supported by the R & D Project in Key Areas of Guangdong (2019B010941002), the Key R & D Project for Social Development in Sichuan (2020YFS0455) and Jiangsu Provincial Key Research and Development Program (CN) (BE2019608).

*Conflict of interest statement*. The authors declare that they have no conflict of interest.
